# Hepatocellular Carcinoma: A Comprehensive Review

**DOI:** 10.3390/diseases13070207

**Published:** 2025-07-02

**Authors:** Nisar Amin, Javaria Anwar, Abdullahi Sulaiman, Nadia Nikolaeva Naumova, Nadeem Anwar

**Affiliations:** 1 Department of Internal Medicine, Charleston Area Medical Center, Charleston, WV 25304, USA; javaria.anwar@vandaliahealth.org; 2 Department of Pathology, Charleston Area Medical Center, Charleston, WV 25304, USA; abdullahi.sulaiman@vandaliahealth.org (A.S.); nadia.naumova@vandaliahealth.org (N.N.N.); 3 Department of Gastroenterology and Hepatology, Charleston Area Medical Center, Charleston, WV 25304, USA; nadeem.anwar@vandaliahealth.org

**Keywords:** HCC, hepatocellular carcinoma, liver cancer, cirrhosis, viral hepatitis, liver cancer etiology, liver cancer treatment

## Abstract

Hepatocellular carcinoma (HCC) is the sixth most common malignancy globally and remains one of the leading causes of cancer-related mortality. Its incidence continues to rise worldwide, and it is currently the fastest-growing cancer by incidence in the United States. HCC most often arises in the context of chronic liver disease, particularly cirrhosis. While chronic viral hepatitis (hepatitis B and C) has traditionally been the primary etiologic factor, recent advances in antiviral therapies and prevention strategies have shifted the epidemiological landscape. Metabolic dysfunction-associated steatotic liver disease (MASLD) and alcohol-related liver disease are increasingly prominent risk factors, especially in Western populations. This shift underscores the need for targeted risk factor modification, improved early detection, and enhanced surveillance protocols. The management of HCC necessitates a multidisciplinary approach, incorporating locoregional therapies, surgical resection, liver transplantation, and systemic therapies for advanced-stage disease. Recent advances in systemic treatments, including immune checkpoint inhibitors and combination therapies, have transformed the therapeutic landscape. Despite these developments, significant challenges persist in optimizing treatment, identifying predictive biomarkers, and personalizing therapy. Ongoing research is focused on refining molecular classifications and advancing precision medicine strategies to improve outcomes. This review provides a comprehensive overview of the etiology, surveillance strategies, diagnostic approaches, molecular features, and current treatment modalities for HCC.

## 1. Introduction

Hepatocellular carcinoma (HCC) is the most common primary liver cancer, accounting for 75–85% of cases, followed by intrahepatic cholangiocarcinoma, which comprises 10–15% of [1] cases. HCC is the sixth most common cancer globally and the third leading cause of cancer-related mortality with a five-year survival rate of only 15% [1,2]. In the United States, the incidence of HCC has tripled since 1980, making it the fastest-growing tumor in the country. HCC-related mortality increased by 43% between 2000 and 2016 [3,4].

HCC primarily arises in the setting of chronic liver disease and cirrhosis, with only a small percentage of cases occurring in individuals without underlying liver pathology [5,6]. Approximately 80–90% of HCC patients have pre-existing cirrhosis, and the five-year cumulative risk of HCC development in cirrhotic patients ranges from 5% to 30%, depending on underlying etiology, severity of cirrhosis, ethnicity, and geographic region [7]. HCC is the leading cause of mortality in patients with cirrhosis [8].

The primary risk factors for HCC include chronic hepatitis B virus (HBV) and hepatitis C virus (HCV) infections, alcohol-related liver disease, and metabolic dysfunction-associated steatotic liver disease (MASLD), all of which play a significant role in the development of cirrhosis and subsequently HCC [9,10,11,12,13]. Other less prevalent causes of cirrhosis that can also lead to HCC include primary biliary cirrhosis, hereditary hemochromatosis, α1-antitrypsin deficiency, smoking, and aflatoxin B1 exposure [14,15,16]. Conversely, several studies have demonstrated a protective effect of coffee and aspirin in reducing the risk of HCC development [17,18,19].

The incidence and prevalence of HCC are rising worldwide, and the World Health Organization (WHO) projects that more than 1 million patients will die from liver cancer by 2030 [20]. This rising trend underscores the urgent need for early detection, improved surveillance, and targeted therapies to mitigate disease progression and mortality.

## 2. Pathogenesis and Genetics

The cellular origin of HCC remains debated, with some studies supporting hepatocytes, while others suggest liver stem cells as progenitors. The pathogenesis of HCC is a multifactorial process involving genetic, epigenetic, and environmental factors that drive malignant transformation. Persistent hepatocyte injury caused by infections or toxins induces chronic inflammation, which in turn triggers aberrant regeneration and fibrosis, ultimately leading to cirrhosis [21]. Over time, cirrhotic liver tissue undergoes progressive genetic and epigenetic alterations, forming dysplastic nodules that evolve into preneoplastic lesions and, ultimately, HCC [20].

HCC is among the cancers with the fewest somatic mutations. The most frequent genetic alteration in HCC is the telomerase reverse transcriptase gene (TERT) promoter mutation (60%), which leads to increased telomerase expression, resulting in telomere shortening and cellular instability, ultimately contributing to HCC development [22]. The TERT gene plays a pivotal role in HCC pathogenesis, particularly in the presence of risk factors such as aflatoxin exposure, smoking, alcohol consumption, and chronic HBV infection [23]. Other frequently mutated genes include TP53 (25%) and CTNNB1 (30%) [24,25]. Next-generation sequencing studies have identified additional cell cycle control gene mutations, including CCNE1, PTEN, CCNE2, ARID1A, ARID2, RPS6KA3, RB1, AXIN1, APC, CCND1, FGF19, VEGFA, MYC, MET, and NFE2L2 [26].

Polymorphisms in MBOAT7, PNPLA3, GSTT1, TM6SF2, and HSD17B13 are associated with hepatic fat accumulation and inflammation, predisposing individuals to cirrhosis and HCC [27,28]. Molecular pathways implicated in HCC pathogenesis include Wnt/β-catenin, PI3K/AKT/mTOR, and MAPK signaling. Approximately 25% of HCCs harbor potentially actionable mutations, making oncogenes and tumor suppressors within these pathways promising therapeutic targets, some of which are currently being investigated in clinical trials [29,30].

Hoshida et al. demonstrated that there were three distinct transcriptomic patterns identified with HCC tumors, and these were named as S1, S2 and S3 [31]. They had distinct activation/signaling pathways with differences in clinical course and tumor biology. S1 tumors follow the Wnt/TGF-beta pathways (independent of Beta-catenin activation), and are associated with worse clinical course. S2 tumors follow the activated Myc/AKT pathways, associated with the larger sized lesions that are AFP-producing tumors. S3 related HCCs are well-differentiated tumors, with a less aggressive clinical course. This makes HCC a very heterogenous type of malignancy, which possibly could explain a variation in response to treatment and also make allowances for a customized treatment plan based on the genetic composition.

## 3. Risk Factors for HCC

### 3.1. Viral Infections

HBV and HCV infections increase the risk of HCC by 20–30 times compared to uninfected individuals [32]. Hepatitis B infection is the most significant risk factor for HCC, accounting for approximately 50% of cases worldwide and remaining the leading cause of HCC in East Asia and most African countries [33,34]. HBV is a DNA virus that causes liver disease by infecting hepatocytes and integrating its DNA into the host genome, leading to cirrhosis and HCC. Additionally, although rare, HBV can directly induce oncogene activation, contributing to HCC development even in the absence of cirrhosis [35].

HBV DNA integrates into specific genomic sites, with the TERT promoter being the most common insertion site, followed by the CCNE1 and KMT2B oncogenes. While most of these integrations are biologically inert, a minority contribute to oncogenesis and the development of HCC. Genetic polymorphisms in these genes are associated with an increased risk of HCC in chronic HBV carriers and may contribute to a more aggressive disease course [36,37].

Similarly, HCV is a major risk factor for liver disease and cirrhosis and significantly increases the risk of HCC. While HBV remains the leading cause of HCC globally, HCV is the primary cause of HCC in the United States and is responsible for the rising prevalence of the disease in the country. Unlike HBV, HCV is an RNA virus and does not integrate into the host genome. Therefore, the primary mechanism of HCC development in HCV-infected individuals is through progressive fibrosis and cirrhosis, with the risk closely linked to the duration of infection [38]. However, studies implicate HCV in epigenetic modifications contributing to hepatocarcinogenesis, primarily through aberrant activation of the Wnt/β-catenin signaling pathway. Mechanisms include HCV core protein–induced promoter methylation and inactivation of tumor suppressors such as SFRP and DKK1, repression of E-cadherin expression, and nonstructural protein-mediated activation of PI3K/AKT signaling, leading to GSK3β inactivation—all promoting malignant transformation [39].

The introduction of direct-acting antiviral (DAA) therapy has led to significant advancements in HCV treatment, with patients achieving sustained virological response (SVR), experiencing up to a 76% reduction in HCC risk. However, despite these improvements, patients with HCV and cirrhosis who have achieved SVR remain at high risk for HCC, necessitating ongoing surveillance [40].

Another less common but clinically important virus associated with HCC is hepatitis D virus (HDV). HDV is an RNA virus that requires the hepatitis B surface antigen (HBsAg) for replication, leading to co-infection or super-infection with HBV. HBV/HDV co-infection accelerates progression to cirrhosis and results in a more aggressive disease course compared to HBV mono-infection alone. Studies have demonstrated that acute or chronic HDV co-infection significantly increases the risk of HCC compared to HBV alone [41].

### 3.2. MASLD

Metabolic dysfunction-associated steatotic liver disease (MASLD), formerly known as non-alcoholic fatty liver disease (NAFLD), is a chronic liver disease characterized by hepatic fat accumulation unrelated to alcohol use and is defined by the presence of at least one cardiometabolic risk factor. The accumulation of fatty acids in hepatocytes generates reactive oxygen species (ROS), leading to DNA damage and disruption of DNA repair mechanisms, thereby promoting HCC development. MASLD is strongly associated with metabolic syndrome and diabetes and has emerged as the fastest-growing cause of HCC, particularly in Western countries [42].

Over the past decade, HCC cases attributed to MASLD have increased rapidly, now accounting for 10–20% of HCC cases in the United States. Similar to HBV, MASLD can lead to HCC even in the absence of cirrhosis. Studies indicate that 27% of MASLD patients who developed HCC had previously undiagnosed cirrhosis [34]. Genetic studies have identified polymorphisms in PNPLA3, which are associated with hepatic fat accumulation, hepatic inflammation, and an increased risk of HCC [43,44]. A higher incidence of this single nucleotide variant has been observed in the Hispanic population, which may contribute to the increased prevalence of HCC in this group [45]. These genetic markers offer potential applications in HCC surveillance and targeted therapy strategies.

### 3.3. Alcohol

Heavy alcohol consumption is an independent risk factor for liver disease and cirrhosis, both of which significantly increase the risk of HCC. Alcohol-associated HCC accounts for 15–30% of HCC cases globally. In the United States, the incidence of alcohol-associated HCC is projected to increase from 1.1 to 2.2 per 100,000 people by 2040 [12].

Alcohol consumption particularly amplifies HCC risk in individuals with other underlying risk factors. Studies indicate that patients with both HBV infection and alcohol use disorder (AUD) exhibit a higher incidence of HCC compared to those with HBV infection alone or AUD alone. A Taiwanese study reported that the 10-year cumulative incidence of HCC was 52.8% in patients with both HBV and alcohol use, compared to 39.8% in those with HBV alone and 25.6% in those with alcoholism alone [46].

### 3.4. Toxins

Aflatoxin B1 (AFB1) is a carcinogenic mycotoxin produced by Aspergillus species, usually found in stored grains. It is strongly associated with an increased risk of HCC following chronic exposure. Aflatoxin exposure occurs primarily through consumption of contaminated food and induces p53 tumor suppressor gene mutations, a hallmark of aflatoxin-related HCC [47]. Studies have demonstrated a dose-dependent relationship between aflatoxin exposure and HCC risk, with aflatoxin accounting for approximately 17% of HCC cases globally [14]. Regions of East Asia and sub-Saharan Africa report high exposure rates to aflatoxin, coinciding with a high prevalence of HBV. Studies have demonstrated that aflatoxin and HBV infection exert a synergistic effect, significantly increasing the risk of HCC [48].

### 3.5. Smoking

Studies indicate that smoking increases the risk of HCC through multiple mechanisms, including interactions with genetic polymorphisms and the introduction of carcinogens that form DNA adducts [49]. This risk is further amplified in individuals with chronic HBV infection and those exposed to aflatoxins [50]. One study reported that smoking more than 25 cigarettes per day was associated with a 55% increased risk of HCC [51]. A detailed table of risk factors is provided in Table 1.

## 4. Epidemiology

The risk of developing HCC varies based on etiology, age, sex, geographic region, and the extent of liver damage. Adults over the age of fifty have an increased risk of developing HCC, with the highest age-specific incidence observed in individuals over 70 years old. Men are at a significantly greater risk than women, with a global male-to-female ratio ranging from 2:1 to 4:1 [32]. The reasons for this disparity are not fully understood but may be attributed to the higher prevalence of risk factors such as chronic HBV and HCV infections, alcohol abuse, and smoking, which are more common in men. Additionally, genetic and hormonal factors have also been implicated in contributing to this difference [52,53].

**Figure 1:**
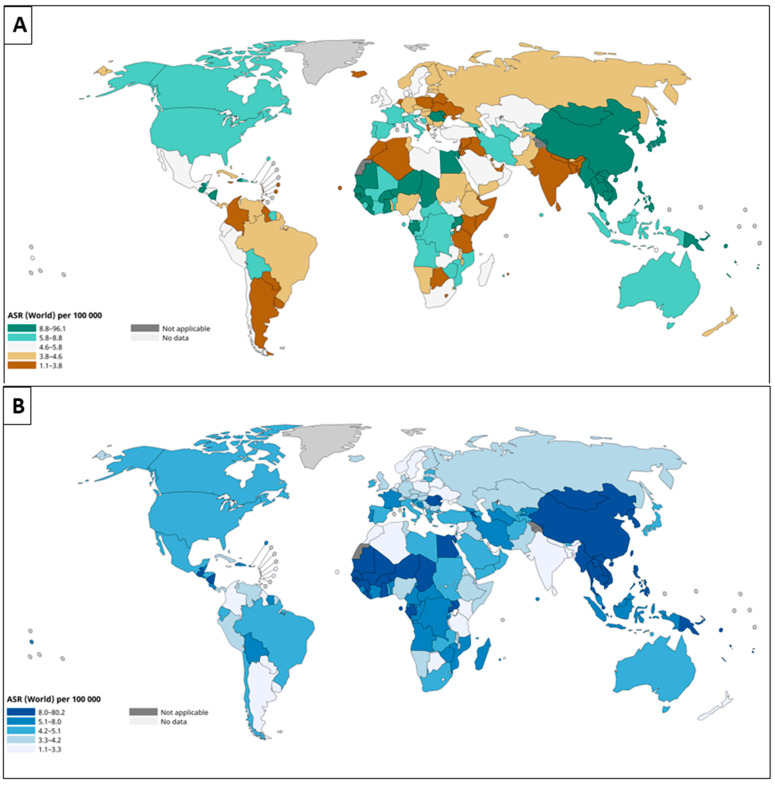
Global geographical distribution of hepatocellular carcinoma. Both incidence (**A**) and mortality (**B**) rates of primary liver cancer are highest in East Asia and sub-Saharan Africa, particularly in regions with limited medical and economic resources. ASR: Age-standardized rates. Data obtained from: https://gco.iarc.fr/today/en/dataviz/maps-heatmap?mode=population&cancers=11&types=1&palette=BrBG&map_nb_color=5. Accessed on 25 April 2025.

The epidemiology of HCC varies significantly across geographic regions. The highest incidence rates are observed in East Asia and sub-Saharan Africa, where HBV infection incidence is highest (Figure 1) [2]. In contrast, North America and Northern Europe have relatively lower HCC incidence rates. However, with the global implementation of HBV vaccination programs and advancements in antiviral therapies, the etiological landscape of HCC is shifting. As a result, a decline in HCC incidence is anticipated in regions with historically high HBV and HCV prevalence [54].

Conversely, HCC incidence is rising in Western countries, primarily due to the increasing prevalence of MASLD and metabolic syndrome. These conditions are expected to become the leading causes of liver disease worldwide owing to the increase in obesity and other metabolic syndromes [55].

## 5. Clinical Features

HCC presents with a variety of clinical features. In its early stages, particularly when confined to the liver, HCC is frequently asymptomatic with clinical symptoms typically emerging as the disease progresses. As the disease advances, patients may develop nonspecific symptoms such as constitutional (B) symptoms, jaundice, anorexia, weight loss, malaise, and upper abdominal discomfort. On physical examination, hepatomegaly and ascites may be evident [56].

In more advanced stages, HCC may metastasize to distant organs such as the lungs, adrenal glands, peritoneum, and bones, producing symptoms specific to the site of involvement. Laboratory evaluation may reveal elevated liver enzymes and bilirubin, often reflecting underlying hepatic dysfunction. Additionally, elevated alpha-fetoprotein (AFP) levels may be observed and can aid in the diagnosis of hepatocellular carcinoma, although this is not universally present. AFP is elevated in up to 70% of cases [57]. Diagnostic imaging, including ultrasound, CT, or MRI, plays a central role in both the diagnosis and staging of HCC, typically revealing hepatic masses with characteristic radiographic features, as described below.

The National Comprehensive Cancer Network (NCCN) guidelines highlight that HCC is often asymptomatic in its early stages and underscores the importance of routine surveillance in at-risk populations to enable early detection and improve treatment outcomes. Given the complexity of HCC, the guidelines also advocate for a multidisciplinary approach to management. This involves a coordinated team of specialists—including hepatologists, diagnostic and interventional radiologists, surgeons, medical oncologists, and pathologists with expertise in hepatobiliary malignancies—to facilitate individualized, evidence-based treatment planning [58].

## 6. Surveillance

Certain high-risk patients are recommended to undergo surveillance for the development of HCC. It has been shown previously that surveillance is cost effective in patients whose risk for development of HCC exceeds 1.5% [59]. These are patients with any form of cirrhosis, those with chronic hepatitis B above the age of 40 and 50 for males and females respectively, and any chronic hepatitis B patients with family history HCC. At present there are no validated scoring systems to accurately predict the risk for HCC.

The most suitable surveillance tool that meets the criteria for cost effectiveness is abdominal ultrasound every 6 months as recommended by American Association for the Study of Liver Diseases (AASLD), the European Association for the Study of the Liver (EASL), and the Asian Pacific Association for the Study of the Liver (APASL). Tzartzeva et al. showed that the combination of US with serum AFP had improved sensitivity in detecting HCC compared to US alone [60]. The authors of this review article recommend a combination of US with AFP, in line with the current AALSD guidelines.

Serum markers other than AFP that are being studied include Lens Culinaris binding subfraction of AFP (AFP-L3%) and des gamma carboxy prothrombin (DCP). Singal et al. showed in 2022 that GALAD score (Gender, Age, AFP-L3%, AFP and DCP) had a sensitivity of up to 72% with a specificity of 90% in detecting HCC [61]. Another more recent study by Dr. El-Serag et al. evaluated another scoring model known as hepatocellular carcinoma early detection score (HES V2.0) and found it to be more effective in HCC diagnosis compared to GALAD score [62].

As more data emerges regarding the efficacy of these biomarkers and the scoring systems, they may become incorporated into the surveillance and diagnosis of HCC in the future. At this time, the AASLD recommends ultrasound and AFP testing every six months as the appropriate surveillance tools.

## 7. Diagnosis

### 7.1. Imaging, Radiomics and AI

The diagnosis of HCC in majority of cases is based on imaging studies. As mentioned above, the initial lesion may be picked up on a surveillance US or the patient may present with any liver-related or unrelated problems and the diagnosis may be made incidentally.

The diagnosis requires a dynamic multi-phase imaging modality, either a CT scan or an MRI with the appropriate contrast material to document the arterial enhancement of the lesion along with the washout in the venous phase of the study. Liver Imaging Reporting and Data System (LI-RADS) score is used to describe the suspicious liver lesions. It is described below (Table 2).

In the above table, Table 2A shows the original LI-RADS scoring and Table 2B shows the revised (and simplified) LI-RADS (rLI-RADS). As noted above, it takes into account not only the enhancement pattern but also other factors like the tumor size, appearance of the capsule and any growth in the lesion over time.

Depending on the LI-RADS status, further follow-up or treatment is determined. LI-RADS 1 is considered as definitely benign, LR-2 is probably benign, LR-3 has an intermediate probability for malignancy, LR-4 is interpreted as probably malignant and LR-5 is considered as definitely malignant. The category LR-M describes metastatic liver lesions, LR-TIV is tumor in vein, and LR-NC is a lesion that is not characterizable [63].

Contrast-enhanced ultrasound (CEUS) is a noninvasive imaging modality that provides real-time visualization of HCC enhancement patterns and is gaining popularity for both diagnosis and follow-up. CEUS utilizes non-iodinated, non-nephrotoxic contrast agents, offering a key advantage in patients with renal impairment. Studies have shown that CEUS demonstrates comparable sensitivity and specificity to contrast-enhanced CT and MRI for the diagnosis of hepatocellular carcinoma. A meta-analysis by Peng et al. demonstrated promising diagnostic performance of CEUS LI-RADS for HCC, with pooled sensitivity of 72% and specificity of 92% [64]. However, a systematic review by Fraquelli et al. found that CEUS missed 22% of HCC cases and led to unnecessary testing in 6% of non-HCC cases [65]). Similarly, in patients with resectable HCC, CEUS misclassified 23% as unresectable and 8% of non-HCC cases underwent resection. A prospective study by Savsani et al. comparing CEUS with CE CT/MRI in assessing residual viable tumor post-TACE found CEUS to have higher sensitivity but lower specificity [66]. While EASL recommends CEUS alongside contrast-enhanced CT and MRI as a first-line diagnostic tool for HCC [67], the AASLD designates CEUS as a second-line option, to be used when CT or MRI are inconclusive, contraindicated, or unavailable—particularly when biopsy is not feasible [63].

Radiomics and artificial intelligence (AI) are emerging technologies with the potential to revolutionize the diagnosis and management of HCC. When integrated with imaging modalities such as MRI, CT, and ultrasound, it can extract subtle imaging features beyond human perception, enhancing lesion detection, characterization, and differentiation [68]. These tools have been utilized to identify biomarkers predictive of immunotherapy response, transarterial chemoembolization (TACE) efficacy, recurrence risk, and overall survival [69,70,71,72,73]. Despite current limitations—including multi-modal data integration, model overfitting, lack of standardization in imaging protocols, limited external validation, and generalizability concerns—ongoing advancements in AI offer significant potential for improving diagnostic precision and supporting personalized treatment strategies in HCC [74].

### 7.2. Pathology

Tissue diagnosis is not required in most cases of HCC if imaging is consistent with the malignant features. However, in LI-4 lesions, as well as in non-cirrhotic patients, such as those with MASLD, a suspicious lesion should be biopsied. However, there is a small but real risk of seeding of the needle track with tumor cells, described as 2.7% in a study done by Silva et al. [75].

**Figure 2:**
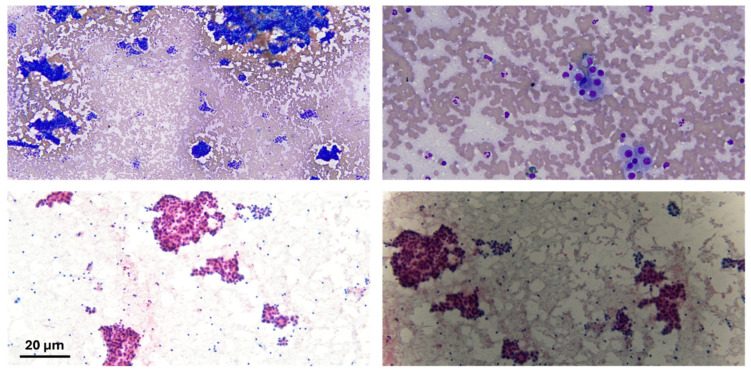
Cytological preparation of lesional material; the lesional material shows atypical sheets of cells in both diff-quick (bright blue) and pap stain (pink cytoplasm). Scale bar: 20 μm.

**Figure 3:**
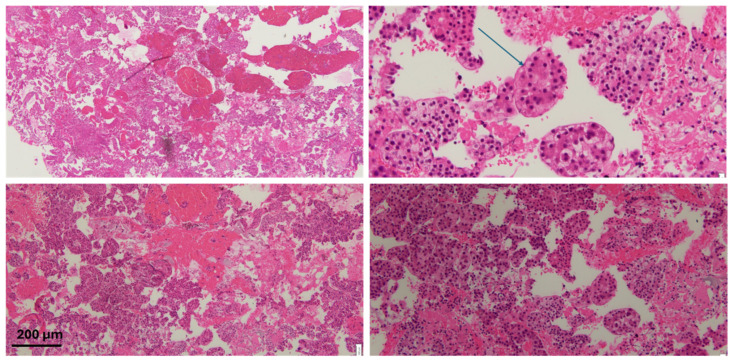
H&E staining of the specimen with atypical cells with some areas of clear/vacuolated cytoplasm. The arrow highlights endothelial wrapping, a feature suggestive of hepatocellular carcinoma. Scale bar: 200 μm.

**Figure 4:**
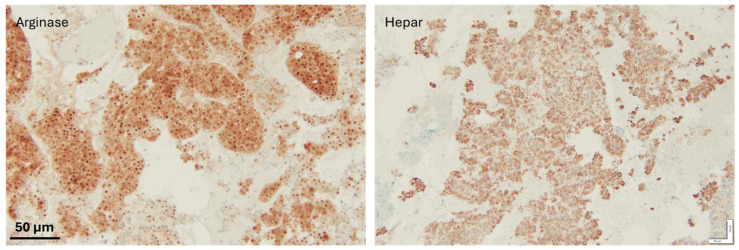
IHC staining to confirm metastasis; both hepatocyte markers were positive, therefore supporting the diagnosis of a metastatic hepatocellular carcinoma. Scale bar: 50 μm.

Typically, the histology will show distortion of the architecture, stromal invasion, unpaired arteries as well as some diffuse fatty changes. Histological staining often reveals an increased nuclear-to-cytoplasmic ratio, nuclear atypia, and a trabecular or psuedoglandular growth pattern (Figure 2 and Figure 3). HepPar-1 and Arginase-1 are frequently employed as immunohistochemical markers of hepatocellular differentiation (Figure 4).

Malignant cells stain positive for heat shock protein-70, glutamine synthetase, glypical-3 and clathrin heavy chain. A ductular reaction can be detected using a CK7/19 stain. Diffuse uptake of CD-34 stain is also seen.

### 7.3. Liquid Biopsy

Liquid biopsy is a minimally invasive technique that uses blood or other body fluids to detect molecular alterations, tumor cells, and metabolites. Key biomarkers include circulating tumor cells (CTCs), circulating tumor DNA (ctDNA), tumor-derived extracellular vesicles (EVs), tumor-educated platelets (TEPs), and circulating free RNA (cfRNA) [76].

In HCC, combining alpha-fetoprotein (AFP) with microRNAs (e.g., miR-221-3p, miR-223-3p, miR-10b-5p, and miR-21-5p) has shown improved early detection, particularly in patients with low AFP levels [77]. Additional protein markers, such as soluble LG3BP and PIGR, are also under investigation for diagnostic and prognostic use [78,79]. ctDNA is being explored for both diagnostic and prognostic purposes, and its whole-genome sequencing has been incorporated into early liver cancer screening programs in China [80].

Liquid biopsy offers notable advantages, including noninvasive sampling and rapid analysis. While many biomarkers remain investigational, ctDNA has received FDA approval in several cancers for early detection, prognostication, and monitoring of recurrence or treatment response [81]. However, the AASLD currently advises against using liquid biopsy for HCC diagnosis outside research settings, pending further validation in large-scale prospective studies [63].

## 8. Treatment

Various treatment options ranging from locoregional therapies to resection to liver transplantation are being utilized in addition to systemic therapy for more advanced cases. The determination of the stage of the tumor involves not only assessing the size and location of the primary tumor(s) but also includes assessment for metastases, the degree of liver dysfunction as well as the functional status of the patient. Hence, it requires a multidisciplinary approach. All cases must be presented to the institutional tumor board so that the best treatment options are considered.

Chest CT scans (non-contrast) should be performed to check for metastases. The patient’s performance status can be assessed using the ECOG scale as shown in the Table 3 [82].

The degree of hepatic dysfunction can be determined by the Childs-Turcotte-Pugh or the MELD score.

There are various staging systems but the one most studied and applied is the Barcelona Liver Clinic Cancer model (BCLC). It was first proposed in 1999 but has been revised in 2022 [83,84]. Based on the above-mentioned parameters, HCC can be divided into very early, early, intermediate, advanced and terminal stages as described below:

1. Very early stage (stage 0): Single lesion < 2 cm, compensated liver disease, ECOG 0.
2. Early stage (stage A): tumor within Milan criteria, ECOG 0, compensated liver disease.
3. Intermediate or Stage B: Tumor outside of Milan criteria, compensated liver disease, ECOG 0.
4. Advanced (stage C): vascular invasion or distal metastases, compensated cirrhosis, ECOG 1-2.
5. Terminal: decompensated liver disease, ECOG 3-4, any size tumor.

## 9. Treatment Options

### 9.1. Ablation

Three different ablation modalities are in use, including radiofrequency (RFA), microwave (MWA) and cryotherapy. Ablation works best for smaller lesions, typically less than 2.5 cm. Livraghi et al. showed that the chance of achieving a complete response for lesions > 5 cm in size was less than 50% [85]. The overall survival rate for any of these modalities when picked appropriately is comparable.

### 9.2. Resection

Surgical resection should be considered for a single lesion in patients with stage 0 or 1 HCC. In non-cirrhotic patients, it can be curative but in patients with cirrhosis, recurrence is high. It can be as high as 50–70% as documented by Cha C et al. [86]. Mlynarsky et al. showed that the 5-year survival after resection ranged between 60 and 80% [87]. Portal pressure assessment is important prior to resection as those with portal hypertension can be at risk for decompensation post resection and should be referred for liver transplantation instead.

### 9.3. Liver Transplantation

For patients with evidence of portal hypertension and tumor within Milan criteria (single tumor less than 5 cm in diameter or three tumors or less with no tumor bigger than 3 cm), liver transplantation offers excellent outcomes. These patients have underlying cirrhosis, which is also treated by this procedure. United Network for Organ Sharing allocates MELD exception points to these patients to allow them to have a timely transplantation before metastases occur. Patients who have tumors outside of the Milan criteria can be successfully transplanted after downstaging them with locoregional therapies like TACE or TARE [88]. Figueras et al. showed that the 5-year survival rate for liver transplantation for HCC was similar to that for non-HCC cirrhosis patients [89].

### 9.4. Trans-Arterial Therapies

The blood supply to the HCC arises from the hepatic artery, a fact that is the basis of the development of transarterial delivery of treatment for the intermediate stage (B) hepatocellular carcinoma. Trans-arterial chemoembolization (TACE) and trans-arterial radioembolization (TARE) are being used to downsize tumors outside of the Milan criteria to get patients transplanted [67]. These treatment modalities can also be offered to more advanced stage patients who are not transplant candidates. Post treatment, patients are followed with contrast enhanced CT or MRI studies to look for any enhancing areas in the lesion. These could represent residual tumor and may require repeat treatments.

### 9.5. Systemic Therapies

Chemotherapy is reserved for stage B tumors with bilobar involvement and for stage C lesions. These patients are not eligible for the resection or transplant option and have a life expectancy of less than 2 years. Various classes of pharmacotherapeutic agents have been used in this regard as described below.

1. Tyrosine kinase inhibitors: sorafenib, regorafenib, lenvatinib and cabozantinib.
2. Monoclonal anti-angiogenic antibodies like bevacizumab and ramucirumab.
3. Immune checkpoint inhibitors, including pembrolizumab, nivolumab, atezolizumab and durvalumab. The former two are programmed death 1 (PD1) inhibitors and the latter two are inhibitors of programmed death ligand 1 (PD-L1).
4. Another category of ICIs includes cytotoxic T-lymphocyte-associated protein 4 inhibitors (CTLA4), including ipilimumab and tremelimumab.

Sorafenib was the first medication that was shown to increase the overall survival by three months (SHARP trial) [90]. The other TKIs were developed later; the last to be approved was cabozantinib [91].

In 2020, the IMBRAVE trial was concluded, evaluating the combination of bevacizumab and atezolizumab compared to sorafenib-based therapy with significantly increased overall survival benefit [92]. Recently, Melero et al. released the 5-year data from CheckMate 040 study evaluating the combination of nivolumab and ipilimumab, showing an objective response rate of 34%, duration of response of 51.2% and the median overall survival of 22 months [93]. The Himalaya study using durvalumab + tremelimumab combination showed a median overall survival of 16.4 months, compared to sorafenib monotherapy (13.8 months) and objective response rate of 20.1 (compared to 5.1) [94]. For patients with high risk for GI bleed, where VEGF inhibitors may pose a risk, this combination treatment can be considered as alternative therapy.

### 9.6. Treatment Recommendations

For very early-stage tumors, local therapy including ablation is recommended. For patients in this category, if they are cirrhotic with normal portal pressures, resection is also an option. If the portal pressures are high, transplant should be considered.

The same recommendations regarding resection vs. transplant are applicable to early stage (stage A) tumor patients. If any contraindications to liver transplantation are identified, ablation can be offered.

For intermediate stage (B), trans-arterial therapies with chemo-embolizations (TACE) or radioembolization (TARE) can be offered. If that can help downsize the tumor into the Milan criteria limits, such patients can be successfully transplanted provided there are no other contraindications to liver transplantation [95].

Patients with diffuse bilobar tumor (stages B or D) have a life expectancy of less than two years and should be offered systemic treatment.

Stage D tumors have a life expectancy of three months or less and should be considered for supportive care and hospice [63].

### 9.7. Future Direction

MicroRNAs (miRNAs) are being studied for their various effects on the disease course of HCC, including cancer cell survival and replication, modification of apoptosis, autophagy, and metastases of cancer cells. Various non-coding RNAs are implicated in tumor development, angiogenesis as well as the tumors’ response/resistance to treatment. Duurland et al. have shown the anti-tumor immunity inferred by INT-1B3 in animal studies [96].

Zhang et al. showed that overexpression of miR-142-3p can increase tumor cell sensitivity to sorafenib [97].

Similarly, miR-214 can target the Wnt pathway and stop mitosis at G1 phase [98]. Upregulation of miR130a-3p can help inhibit tumor growth and metastasis [99]. Phase II trials are ongoing employing chimeric antigen receptor T cells (CAR-T), against CD133 as well as GPCR3 targets [100].

Further research on human trials is still pending but once available, it will make personalized care a reality for the treatment of HCC, based on the genetic profiles of the individual patients. Similarly, work is being carried out on potential cancer vaccines.

## 10. Conclusions

Hepatocellular carcinoma is among the top etiologies for cancer-related deaths worldwide. Effective treatment guidelines must be followed to help physicians make management decisions leading to prevention, early detection, and appropriate treatment as well as surveillance post treatment to prevent recurrence and to minimize disease-related morbidity and mortality.

## Figures and Tables

**Table 1:** Common risk factors for hepatocellular carcinoma (HCC).

Risk Factor	Category	Mechanism
Cirrhosis (any etiology)	Liver Disease	Regenerative nodules, chronic inflammation
Chronic Hepatitis B Virus (HBV)	Viral Infection	Integration into host genome, direct oncogenesis
Chronic Hepatitis C Virus (HCV)	Viral Infection	Chronic inflammation, cirrhosis, fibrosis
Alcohol Use Disorder	Lifestyle	Chronic liver injury, cirrhosis
Metabolic Dysfunction-Associated Steatotic Liver Disease (MASLD)	Metabolic/Liver Disease	Lipotoxicity, inflammation, progression to cirrhosis
Aflatoxin B1 Exposure	Environmental	DNA adduct formation (e.g., p53 mutations)
Obesity	Metabolic	Associated with MASLD
Smoking	Lifestyle	Carcinogen exposure
Hemochromatosis	Genetic Disorder	Iron overload, oxidative stress
Primary Biliary Cholangitis	Autoimmune Liver Disease	Chronic inflammation, bile duct damage
α1-Antitrypsin Deficiency	Genetic Disorder	Abnormal ATT protein accumulation, cirrhosis
Male Sex	Demographic	Possibly hormonal; epidemiological association
Older Age	Demographic	Cumulative exposure to risk factors
Family History of HCC	Genetic/Environmental	May reflect shared genes or exposures

**Table 2:** LI-RAD scoring system original (**A**) and revised (**B**).

(**A**)				
**APHE**	**Size (mm)**	**0 Additional Features**	**1 Additional Feature**	**≥2 Additional Features**
None	<20	LR-3	LR-3	LR-4
	≥20	LR-3	LR-4	LR-4
Nonrim APHE	<10	LR-3	LR-4	LR-4
	10–19	LR-3	LR-4/LR-5 *	LR-5
	≥20	LR-4	LR-5	LR-5
(**B**)				
**APHE**	**Size (mm)**	**0 Additional Features**	**1 Additional Feature**	**≥2 Additional Features**
None	Any size	rLR-3	rLR-4	rLR-5
Nonrim APHE	<20	rLR-3	rLR-5	rLR-5
	≥20	rLR-4	LR-5	LR-5

Major features include enhancing “capsule”, non-peripheral “washout”, and threshold growth. * If enhancing “capsule” (LR-4), non-peripheral “washout”, or threshold growth (LR-5). Table 2 was created using data from the AASLD 2023 guidelines [63].

**Table 3:** ECOG performance status scale.

Grade 0	Full level of activity with no limitations
Grade 1	Ability to perform activities of light/sedentary nature
Grade 2	Capable of performing activities of daily living but not able to work, ambulatory for >50% of waking time
Grade 3	Limited self-care, bed- or chair-bound for >50% of waking time
Grade 4	Completely confined to bed or chair
Grade 5	Pt deceased
