# Correction: Amin et al. Hepatocellular Carcinoma: A Comprehensive Review. *Diseases* 2025, *13*, 207

**DOI:** 10.3390/diseases13080257

**Published:** 2025-08-13

**Authors:** Nisar Amin, Javaria Anwar, Abdullahi Sulaiman, Nadia Nikolaeva Naumova, Nadeem Anwar

**Affiliations:** 1 Department of Internal Medicine, Charleston Area Medical Center, Charleston, WV 25304, USA; 2 Department of Pathology, Charleston Area Medical Center, Charleston, WV 25304, USA; abdullahi.sulaiman@vandaliahealth.org (A.S.);; 3 Department of Gastroenterology and Hepatology, Charleston Area Medical Center, Charleston, WV 25304, USA; nadeem.anwar@vandaliahealth.org

## 1. Table Legend

In the original publication [1], there was a mistake in the legend for Table 2. Table 2 did not include the data source in the table footer. The correct legend appears below.

Major features include enhancing “capsule”, non-peripheral “washout”, and threshold growth. * If enhancing “capsule” (LR-4), non-peripheral “washout”, or threshold growth (LR-5). Table 2 was created using data from the AASLD 2023 guidelines [63].

## 2. Error in Figures 2–4

In the original publication, there was a mistake in Figures 2–4 as published. Figures 2–4 lacked scale bars and corresponding descriptions in their legends. The corrected versions of Figure 2, Figure 3 and Figure 4 appear below.

## 3. Missing Institutional Review Board and Informed Consent Statement

In the original publication, the Institutional Review Board and Informed Consent Statement were not included.

**Institutional Review Board Statement:** The histological images (Figures 2–4) were obtained from de-identified human tissue samples. As no identifiable patient information is included, this study was exempt from IRB review in accordance with institutional guidelines.

**Informed Consent Statement:** Histologic images were used in accordance with institutional regulations. Per institutional policy, informed consent was not required for the use of de-identified pathology images.

The authors state that the scientific conclusions are unaffected. This correction was approved by the Academic Editor. The original publication has also been updated.

## Figures and Tables

**Figure 2:**
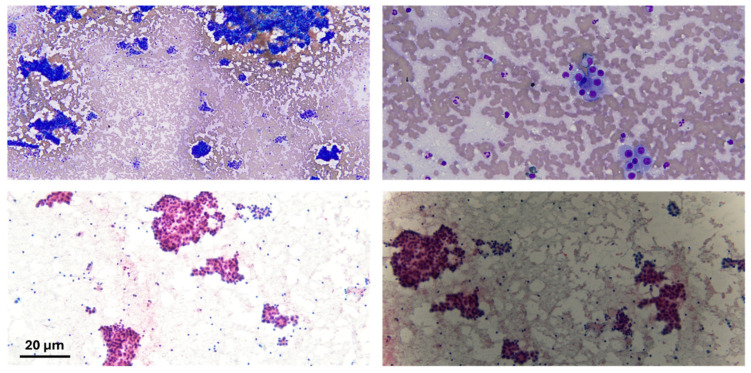
Cytological preparation of lesional material; the lesional material shows atypical sheets of cells in both diff-quick (bright blue) and pap stain (pink cytoplasm). Scale bar: 20 µm.

**Figure 3:**
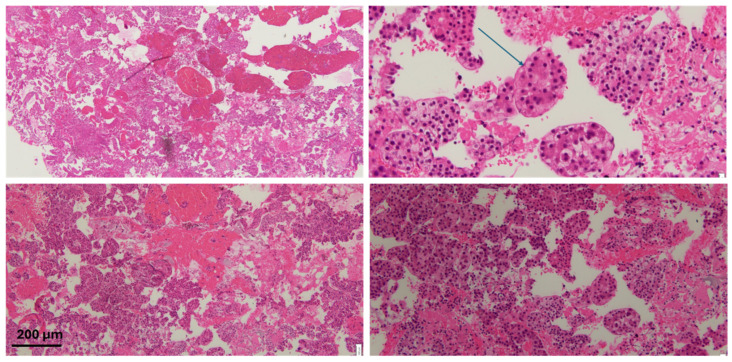
H&E staining of the specimen with atypical cells with some areas of clear/vacuolated cytoplasm. The arrow highlights endothelial wrapping, a feature suggestive of hepatocellular carcinoma. Scale bar: 200 µm.

**Figure 4:**
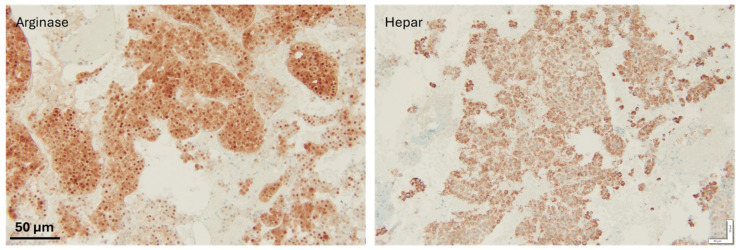
IHC staining to confirm metastasis; both hepatocyte markers were positive, therefore supporting the diagnosis of a metastatic hepatocellular carcinoma. Scale bar: 50 µm.
